# Tandem-structured, hot electron based photovoltaic cell with double Schottky barriers

**DOI:** 10.1038/srep04580

**Published:** 2014-04-03

**Authors:** Young Keun Lee, Hyosun Lee, Jeong Young Park

**Affiliations:** 1 Center for Nanomaterials and Chemical Reactions, Institute for Basic Science, Daejeon 305-701, South Korea; 2 Graduate School of EEWS and NanoCentury KI, KAIST, Daejeon 305-701, South Korea

## Abstract

We demonstrate a tandem-structured, hot electron based photovoltaic cell with double Schottky barriers. The tandem-structured, hot electron based photovoltaic cell is composed of two metal/semiconductor interfaces. Two types of tandem cells were fabricated using TiO_2_/Au/Si and TiO_2_/Au/TiO_2_, and photocurrent enhancement was detected. The double Schottky barriers lead to an additional pathway for harvesting hot electrons, which is enhanced through multiple reflections between the two barriers with different energy ranges. In addition, light absorption is improved by the band-to-band excitation of both semiconductors with different band gaps. Short-circuit current and energy conversion efficiency of the tandem-structured TiO_2_/Au/Si increased by 86% and 70%, respectively, compared with Au/Si metal/semiconductor nanodiodes, showing an overall solar energy conversion efficiency of 5.3%.

There has been substantial research focused on converting solar energy into electricity since the development of the pn-junction solar cell. Recently, various strategies have been proposed to enhance the maximum possible efficiency of a solar cell with a band gap of 1.1 eV, 30% of which is called the single-junction Shockley–Queisser limit^1^. Semonin et al. demonstrated photocurrent enhancement assisted by multiple exciton generation on semiconductor quantum dots, which led to a quantum efficiency exceeding 100% in the quantum dot solar cell^2^. The plasmonic solar cell could be a viable thin solar cell capable of efficiently absorbing broad light wavelengths. As the thickness of the photoactive materials increases, light harvesting is improved, whereas carrier recombination easily occurs, leading to a trade-off between light absorption and carrier collection. For excellent optical absorption without considerable loss of charge carriers in a thin solar cell, the surface plasmon based solar cell has been studied. Dang et al. observed improved broadband light harvesting when using multiple core–shell structured oxide/metal/oxide plasmonic nanoparticles in a dye-sensitized solar cell (DSSC), resulting in an increased photovoltaic cell efficiency (PCE) from 8.3% to 10.8%^3^. Atwater et al. and Reineck et al. have suggested possible mechanisms for enhancement of solar cell performance based on plasmonic materials^4^,^5^. There are possibilities for implementation of metal nanoparticles in solar cells that have local near-field enhancement, a high scattering cross section, and an actual light harvesting element that induces charge separation. In addition, tunable surface geometry that increases the surface area for higher dye loading and light harvesting and reduced charge recombination in DSSC were reported by Ko et al. who fabricated a nanoforest composed of high-density, long-branched, multi-generation hierarchical ZnO nanowires^6^. However, an intrinsic challenge in solar cells is limited light absorption on the photoactive materials. A stacking structure generally consisting of a low-bandgap material and a high-bandgap material—called a tandem cell—was introduced to maximize absorption across the solar spectrum^7^. Recently, application of tandem cells has expanded from an inorganic solar cell to an organic solar cell that includes a polymer solar cell to overcome low carrier mobility (i.e., a limiting factor for power conversion efficiency)^8^,^9^,^10^. Dou et al. designed a tandem polymer solar cell that consists of a low-bandgap conjugated polymer spectrally matched with the front cell, showing an increased PCE (8.62%), compared with a single-layer polymer solar cell (6%)^11^.

The initial effort to develop a solar energy conversion device can be traced back to the metal/semiconductor (MS) junction with a thin metal film. The MS diode has two mechanisms for generation of excited electrons as photocurrent: band-to-band excitation on the semiconductor and internal photoemission from the thin metal film (i.e., hot electrons). It is known that hot electrons, which have high kinetic energy (1–3 eV), can be generated in metals when external energy is deposited on the surface, such as in the absorption of light^12^. Energetic charge carriers excited during photon illumination have been detected on thin metal/semiconductor contacts or metal/insulator/metal tunneling junctions^13^,^14^,^15^. It was reported that surface modification of a thin metal film, such as a connected island structure or deposited nanowires, enhanced the yield of energetic charge carrier generation. Knight et al. showed the possibility for photodetection using energetic charge carriers injected over a Schottky energy barrier at the interface between a gold nanostructure (the antenna) and n-type silicon^16^. The gold nanostructure was coupled with incident photons and exhibited resonant plasmons that excited energetic charge carriers and resulted in a detectable photocurrent^17^,^18^. Wang et al. observed excitation of hot carriers via plasmon absorption on Au/Al_2_O_3_/Au junctions, suggesting that a simple planar metal/insulator/metal diode can play a role as a light harvesting device based on hot electron extraction^19^. Among approaches to increase the PCE in MS solar cells based on internal photoemission and band-to-band excitation, parallel energy harvesting with tandem cells could be ideal.

In this work, we demonstrate solar energy conversion using a tandem-structured, hot electron based photovoltaic cell with double Schottky barriers for multiple reflections of hot electrons and two semiconductor layers for enhancement of band-to-band excitation, as illustrated in Fig. 1a. We show a tandem-structured solar cell composed of multilayers with different absorption characteristics, which is a promising strategy to increase energy conversion efficiency.

## Results and discussion

**Figure 1:**
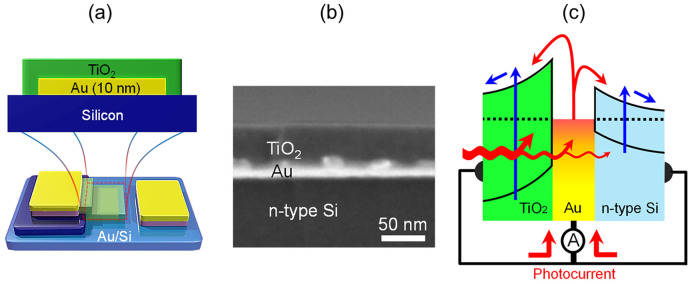
(a) Scheme of a tandem-structured TiO_2_/Au/Si diode where the TiO_2_ layer is stacked as the second semiconductor on a thin gold film to form double Schottky barriers. (b) A cross-sectional image of the TiO_2_-deposited Au film on silicon, showing an approximately 50 nm thick layer of TiO_2_ and a 10 nm thick layer of Au on the silicon. The Au layer plays a role as a common electrode to the two semiconductors and as an optical absorption layer for internal photoemission. (c) The energy band diagram for internal photoemission enhanced by the double Schottky barriers and band-to-band excitation on the two semiconductors. The red line represents internal photoemission and generation of hot electrons on the metal and the blue line represents separation of the electron/hole pairs on two semiconductors by band-to-band excitation.

In the tandem-structured, hot electron based photovoltaic cell, the double Schottky barriers were built on the two interfaces of a thin gold film that makes contact with the TiO_2_ and silicon. Fig. 1b shows a cross section of TiO_2_ deposited on a Au film on silicon, where the Au layer plays a role as a common electrode that has Schottky contacts to two semiconductors. The corrugated Au layer (periodic pattern) refers to the cross-section view of the Au island structures. The Au layer is also an optical absorption layer for internal photoemission. The transport process of electrons on a metal/semiconductor contact is illustrated in the energy diagram in Fig. 1c. The proposed tandem-structured, hot electron based photovoltaic cell harvests the photo-excited charge carriers via two channels: band-to-band excitation on two semiconductors and internal photoemission (or hot carrier generation) based on absorption on the metal layer and transport across the double Schottky barriers.

Two pathways for internal photoemission and band-to-band excitations are shown as the red and blue lines in Fig. 1c, respectively. As the incident photon energy (*hν*) is greater than the band gap (*Eg*) of the semiconductors, electron–hole pairs are produced in the semiconductors while hot electrons (via the internal photoemission process) are generated by absorption of incident photon energy on the thin gold film. In the case where the Schottky energy barrier (*E_SB_*) < *hν* < *Eg*, the predominant route to excite energetic electrons is internal photoemission in the metal film because the band-to-band excitation of the semiconductors can be negligible in the photon energy range^13^,^20^. The total photocurrent (*A*) measured on the tandem structure can be obtained by collecting the photocurrent from both subcells, as shown in Fig. 1c. Christine et al. developed models of the effect of the Schottky barrier on internal photoemission from the metal film into the semiconductor^21^. They presented the enhanced emission probability of hot carriers in the case of double Schottky barriers as a result of multiple hot carrier reflections within the metal film and emission over two Schottky barriers, compared with a single Schottky barrier. In our study, we combined these two mechanisms using a tandem-structured Schottky diode with double Schottky barriers. We found that the tandem-structured Au/Si diode capped with a TiO_2_ layer as the second semiconductor exhibited improved solar energy conversion efficiency because of an increased incident photon to current conversion efficiency (IPCE) in the visible light region.

**Figure 2:**
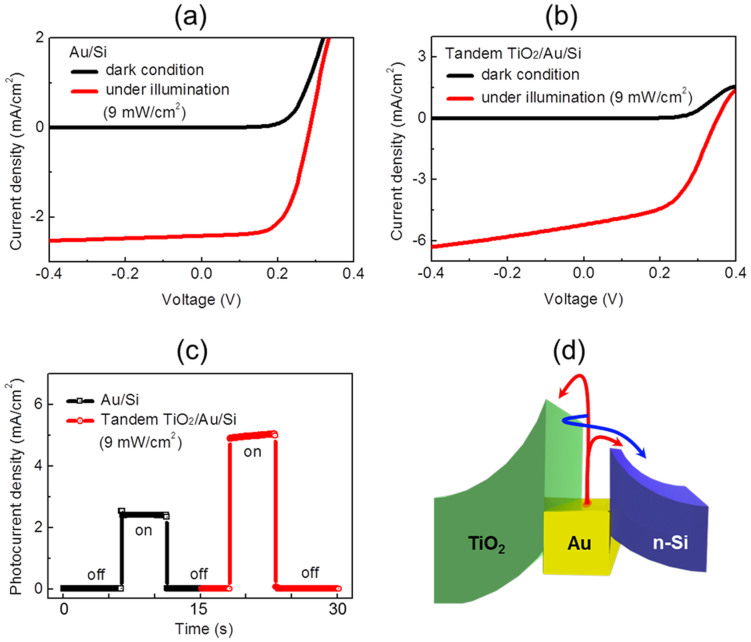
Current–voltage curves measured on (a) the Au/Si diode and (b) the tandem-structured TiO_2_/Au/Si diode, showing an enhanced light response under illumination by a halogen–tungsten lamp (9 mW/cm^2^). The current–voltage curves without light illumination reveal that the rectifying behaviors of the diodes were well maintained. (c) Plot of a short-circuit photocurrent measured on the Au/Si diode and the tandem-structured TiO_2_/Au/Si diode. (d) Energy band diagram for the effect of double Schottky barriers leading to multiple reflections of hot electrons, resulting in enhanced internal photoemission.

The effect of the double Schottky barriers on photocurrent was observed on the TiO_2_-deposited Au/Si diodes. Fig. 2a,b shows current–voltage curves of Au/Si and TiO_2_/Au/Si, respectively, revealing that the photocurrent increased when illuminated by a tungsten–halogen lamp and that the rectifying property of the diodes was well preserved after deposition of TiO_2_ on the Au/Si. The photocurrent density measured under illumination by a halogen–tungsten lamp dramatically increased from 2.4 to 5.0 mA/cm^2^ after deposition of TiO_2_ on the Au/Si (Fig. 2c). While the Au/Si cell shows a rectangular shape, as expected, the TiO_2_/Au/Si tandem cell exhibited transient behavior (2% increase in current for 5 seconds). Such transient behavior was recently found in TiO_2_-based heterojunctions and was attributed to dielectric effects and oxide instability^22^.

The energy band diagram of the TiO_2_-deposited Au/Si diodes could be different from that of Au/Si, as proposed by Fig. 2d, because the second Schottky barrier was built at the interface between the TiO_2_ and the thin gold layer. Fig. 2d shows the routes for internal photoemission from the thin gold film to both semiconductors through the double Schottky barriers. First, hot electrons with energy higher than each Schottky energy barrier can overcome both Schottky barriers toward the semiconductors (red lines). In addition, the emission probability of the hot electrons can be enhanced through multiple reflections (blue line) until the excess energy of the hot electrons is reduced to the lower Schottky energy barrier. If the photons with energy *hν* are absorbed uniformly and not emitted, the hot electrons are assumed to reflect elastically and diffusely from the metal/semiconductor interfaces. The number of trips taken by the hot electrons via multiple reflections within the double Schottky barriers can be given by 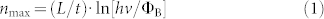
where *n* is the total number of trips the hot electrons take via multiple reflections, *L* is the mean free path of the hot electrons, *t* is the thickness of the thin metal film, *hν* is the photon energy, and Φ_B_ is the Schottky barrier^21^. In the case of a single Schottky barrier, the value of *n*_max_ decreases by half because there is only one route for internal photoemission. At the same time, improved efficiency of the band-to-band excitation is expected by absorption of photon energy on the two semiconductors with different band gaps. In the tandem-structured Au/Si, the anti-reflecting effect can be negligible because the refractive index of the thin gold film is smaller than that of the TiO_2_ film in the range of visible light incident on the tandem-structured Au/Si^23^.

**Figure 3:**
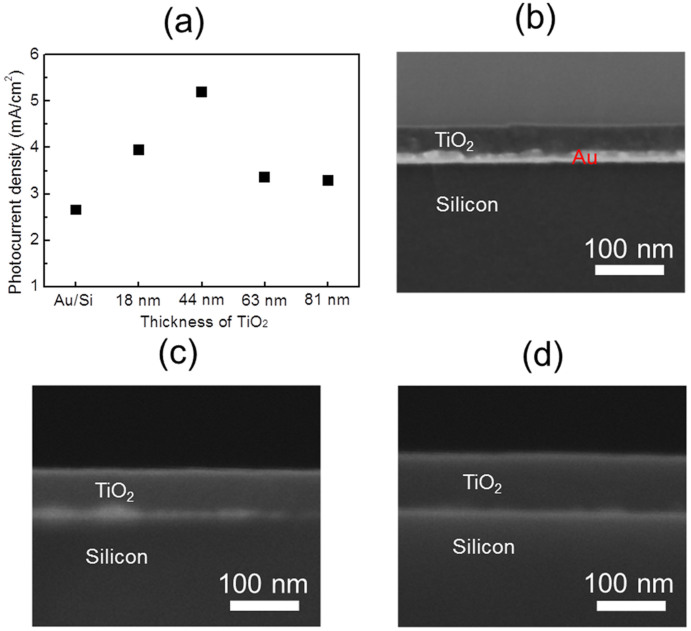
(a) Plot of photocurrent density as a function of TiO_2_ layer thickness under light illumination. Cross-sectional SEM images of (b) a 44 nm thick TiO_2_ layer, (c) a 63 nm thick TiO_2_ layer, and (d) a 81 nm thick TiO_2_ layer.

The photocurrent density was measured as a function of TiO_2_ layer thickness (Fig. 3a). The thickness of the TiO_2_ was controlled by changing the concentration of the sol–gel solution, as shown in Fig. 3b–d. The photocurrent density increased as the TiO_2_ layer thickness increased up to 44 nm thick; however, the photocurrent density decreased when the TiO_2_ layers were thicker than 44 nm. An appropriate TiO_2_ thickness is required to guarantee band bending at the interface between the TiO_2_ and the Au. In the case of the 22 nm thick TiO_2_ layer, the TiO_2_ layer could be too thin for stable band bending at the interface between the TiO_2_ and the Au. On the other hand, when the TiO_2_ thickness exceeds 44 nm, absorption of incident light on the TiO_2_ increases, leading to a decrease of incident light to the Au and Si. Therefore, there exists an optimal thickness (44 nm) of TiO_2_ to maximize the photocurrent in the tandem-structured TiO_2_/Au/Si diode.

**Figure 4:**
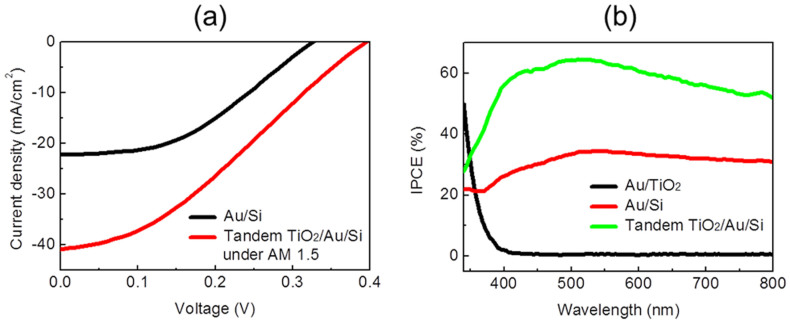
(a) Current–voltage characteristic for solar cell performance under AM 1.5 G. In the tandem-structured TiO_2_/Au/Si, the short–circuit current and the open–circuit voltage increased, compared with those of Au/Si, whereas the fill factor was smaller than that of Au/Si because the resistance factor increased. (b) Incident photon to current conversion efficiency as a function of wavelength measured on the Au/Si diode (red line) and the tandem-structured TiO_2_/Au/Si diode (green line), showing that the tandem-structured TiO_2_/Au/Si exhibited an enhanced efficiency, compared with that of Au/Si, across the entire range of wavelengths. The black line represents the IPCE of Au/TiO_2_, revealing no generation of photocurrent under visible light.

Fig. 4a shows solar cell performance characteristics of the tandem-structured Au/Si. The short-circuit current density (*J_SC_*) and open-circuit voltage (*V_OC_*) were measured on the Au/Si (black line) and the tandem-structured TiO_2_/Au/Si (red line) diodes under illumination of air mass 1.5 global (AM 1.5 G). After TiO_2_ deposition, the *J_SC_* increased from 22 to 41 mA/cm^2^, showing that the tandem-structured metal/semiconductor diode has an improved capability to harvest photon energy by utilizing the double Schottky barriers. The *V_OC_* increased from 0.33 V to 0.40 V. These *V_OC_* values are rather low, comparable to a diazole-based cell^24^. A possible explanation for the increased open-circuit voltage after the adsorption of TiO_2_ on the Au/Si could be associated with a change in the Schottky barrier height. The change in the Schottky barrier of the TiO_2_-adsorbed Au/Si, compared with the Au/Si, was obtained by fitting I–V curves to the thermionic emission equation (Fig. S1). The Schottky barrier height was modified from 0.816 eV to 0.842 eV. In this approach, the Schottky barrier height, 0.842 eV, is representative of the overall value of the combined double Schottky barriers. For an ideal Schottky contact, the Schottky barrier height is expected to be Φ_B_ = Φ_m_ − χ, where Φ_m_ is the work function of the metal and χ is the electron affinity of the semiconductor (n-type)^20^. In the Schottky diode, the *V_OC_* is linearly proportional to the Schottky barrier height^25^. In the double Schottky barrier, the overall Schottky barrier height can be affected by the formation of the second Schottky barrier because electrons overcome the potential barrier and travel into the semiconductors from the metal film in the thermionic emission process. Therefore, if a higher Schottky barrier is formed at the interface of the TiO_2_ and the Au film rather than at the interface of the silicon and the Au film (0.816 eV), the overall Schottky barrier height can become higher (0.842 eV). In our previous work, the experimental Schottky barrier height of ~1.1 eV for Au/TiO_2_ was reported, which is acceptable compared to the literature value^26^,^27^. As a result, it is probable that the newly formed higher Schottky barrier at the interface of the Au/TiO_2_ could increase the overall Schottky barrier height, resulting in the increased *V_OC_*. Fig. 4b shows the IPCE obtained by measuring photocurrent as a function of wavelength. The IPCE plot of the tandem-structured TiO_2_/Au/Si is greater than that of the Au/Si across the entire range of wavelengths, whereas the Au/TiO_2_ didn't exhibit any photon response in the visible light spectrum. The improved ability for current generation of the tandem structure can originate from multiple reflections between the double Schottky barriers and enhanced band-to-band excitation. In addition, it is possible that the increase in the IPCE plot could be attributed to improved penetration of the incident light through the TiO_2_/Au, enhancing light absorption on the silicon substrate. The absorbance spectra were measured using a UV-Vis spectrometer on the TiO_2_-deposited Au film on quartz (Fig. S2). The deposition of the TiO_2_ film on the Au film improved light penetration to the silicon substrate in the visible range, while the TiO_2_ film on quartz absorbed nearly no incident light.

The current density of the Si/Au/TiO_2_ tandem cell is 41 mA/cm^2^ under AM 1.5 G with a light intensity of 100 mW/cm^2^, which results in a photovoltaic cell efficiency of 5.3% in the hot electron tandem cell. Compared with that of the Au/Si structure (3.1%), the tandem cell exhibited an increase in efficiency of ~70%. The *V_OC_* and *J_SC_* under AM 1.5 G improved on the tandem-structured TiO_2_/Au/Si; however, the fill factor (*FF*) decreased because the resistance factors of the cell increased (Fig. S3)^17^. The dielectric effect of the TiO_2_/Au/Si tandem cell can be at least partly associated with enhancement of the short-circuit current. However, as the change in current is not significant (i.e., less than 2% for 5 seconds), the dielectric effect or instability of the oxide might be minor effects.

**Figure 5:**
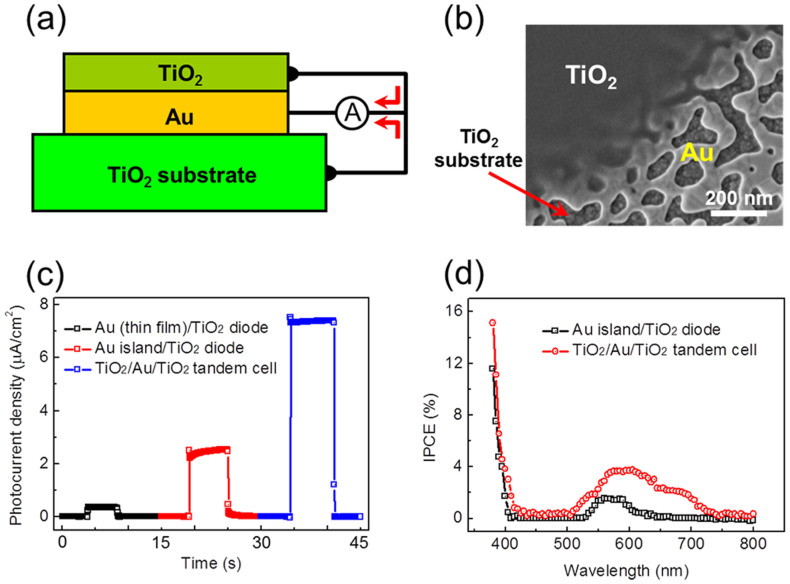
(a) Schematic and (b) SEM image of the TiO_2_/Au/TiO_2_ tandem cell showing the edge of the top TiO_2_ layer on the Au/TiO_2_ diode. (c) Plot of photocurrent density under light illumination measured on the thin film Au/TiO_2_, connected Au island/TiO_2_ diode, and TiO_2_/Au/TiO_2_ tandem cell using a halogen–tungsten lamp (9 mW/cm^2^). (d) IPCE as a function of wavelength measured on the connected Au island/TiO_2_ diode (black line) and the TiO_2_/connected Au/TiO_2_ tandem cell (red line).

To verify the concept of a tandem-structured hot electron solar cell, we constructed a TiO_2_/Au/TiO_2_ tandem cell and compared the photocurrent of the tandem cell with that of a Au/TiO_2_ single cell. Fig. 5a,b shows the scheme and scanning electron microscopy (SEM) image of the TiO_2_/Au/TiO_2_ tandem cell, respectively. The top TiO_2_ layer (50 nm thick) was prepared and coated on a Au/TiO_2_ single cell using the sol–gel method. Fig. S4 shows the representative I–V curves of a Au/TiO_2_ single cell and the TiO_2_/Au/TiO_2_ tandem cell. After surface modification of the thin Au film and deposition of TiO_2_, the rectifying property was well preserved. Fig. 5c shows photocurrent measured on Au/TiO_2_, Au islands/TiO_2_, and TiO_2_/Au islands/TiO_2_. The higher photocurrent was obtained on the Au islands/TiO_2_, compared with the thin film Au/TiO_2_, as a result of surface plasmons^18^. A thin Au film was modified to a connected island structure to enhance hot electron generation via surface plasmons. The photocurrent of the TiO_2_/Au islands/TiO_2_ tandem solar cell was higher than that of the Au islands/TiO_2_ by a factor of three, which is beyond the range of the photocurrent caused by an antireflection layer. As shown in Fig. 5d, the peak in IPCE of the TiO_2_/Au/TiO_2_ originating from hot electron flow is broader and higher than that of the Au/TiO_2_, which could be associated with enhanced hot electron flows because of the double barriers. We note that there is a slight contribution to photocurrent by thermionic emission in the range of 400–520 nm (IPCE < 0.5%); the exact contribution depends on the defect level of the TiO_2_^17^,^18^. In addition, since the metallic layer is surrounded by semiconductor layers with different dielectric constant, the potential difference in the tandem cell can give additional complexities^28^.

This study suggests that the enhancement of hot electron injection by the use of double Schottky barriers is a general phenomenon. The improvement of the solar cell performance suggests that the concept of double Schottky barriers has significant potential as a novel strategy for light harvesting with high energy conversion efficiency.

In summary, hot electron-based tandem cells of TiO_2_/Au/Si and TiO_2_/Au/TiO_2_ were fabricated for enhanced light harvesting. In the tandem-structured cell, the energy band diagram was modified to form double Schottky barriers at both interfaces between the Au film and the semiconductors. The double Schottky barriers can play a role as a reflector for an enhanced hot electron's trip and as an additional route for internal photoemission. In addition, light absorption is improved by the band-to-band excitation of both semiconductors with different band gaps. The effect of the double Schottky barriers was exhibited through enhanced performance of the solar cell. After modification from Au/Si to the tandem-structured TiO_2_/Au/Si, the *J_SC_* and solar cell efficiency were enhanced by 86% and 70%, respectively. The photocurrent of TiO_2_/Au/TiO_2_ exhibited a threefold increase, compared with that of the Au/TiO_2_ single cell. A tandem hot electron photovoltaic device with double Schottky barriers may pave the way for novel methods for light harvesting.

## Methods

For fabrication of a tandem-structured Schottky diode, vertically oriented Au/Si Schottky diodes were fabricated on an n-type (100) silicon wafer. The first step consists of depositing a 200 nm thick film of SiO_2_ on a Si wafer via plasma-enhanced chemical vapor deposition through an aluminum shadow mask. A 50 nm thick film of aluminum and a 150 nm thick film of Au are then deposited through a second mask using electron beam evaporation, which constitutes the nanodiode's two ohmic electrodes. Finally, a thin gold film (10 ± 2 nm thick) is deposited through a third mask. The short-circuit photocurrent was measured with a sourcemeter (Keithley Instrumentation, 2400) under illumination from a halogen–tungsten lamp. Only the active area of the diode was illuminated by the light source. We used a metal mask to block the other areas of the device. In addition, we measured the photocurrent using a laser light source (green, 532 nm ± 10, 5 mW) with a beam size of 0.5 mm. We found that when the laser source illuminated the active area, photocurrent was generated. Therefore, we can rule out the contribution of photocurrent from the bulk Si that is outside of the active region.

For electrical characterization of the diodes, current–voltage measurements were performed by sweeping the voltage between two electrodes and measuring the resulting current. By fitting the current–voltage curves of the diodes to the thermionic emission equation, the Schottky barrier heights, ideality factors, and series resistances for the nanodiodes were obtained^20^,^27^.

For preparation of a TiO_2_ film, the sol–gel process was utilized. First, 0.5 ml of titanium tetraisopropoxide (Ti(OC_3_H_7_)_4_), or TTIP, was added to 9.5 ml of ethanol, which was then mixed with 5 μl of nitric acid^29^,^30^. The TTIP and ethanol were used for the precursor and solvent, respectively. The nitric acid assists the TiO_2_ sols to form a gel and prevents precipitation of the TiO_2_ particles. The TiO_2_ was deposited using the spin coating method. Optical properties of the films were characterized using a UV-Vis spectrophotometer (U–4001, Hitachi). The spectral range capability of the instrument is 300–1100 nm. For measurement of the films, the thin gold layer and the sol–gel TiO_2_ were deposited on a 1/16″ thick quartz window, and the instrument was used in standard setup mode. A bare quartz window was used as a reference to remove the absorption of the quartz.

## Author Contributions

Y.K.L. and J.Y.P. planned the projects and designed the experiments; Y.K.L. and H.L. fabricated the devices; all the authors interpreted and discussed the results and commented on the manuscript.

## Supplementary Material

Supplementary Information Supplementary Information
