# Supplementary material for: Tandem-structured, hot electron based photovoltaic cell with double Schottky barriers
Source: Sci Rep. 2014 Apr 3;4:4580. doi: 10.1038/srep04580 (PMC3974133; doi:10.1038/srep04580)
Supplement: Supplementary Information [file srep04580-s1.doc]

Supporting Information for

**Tandem-structured, Hot Electron Based Photovoltaic Cell with Double Schottky Barriers**

Young Keun Lee^1,2^, Hyosun Lee^1,2^ and Jeong Young Park^1,2^,*

*^1^Center for Nanomaterials and Chemical Reactions, Institute for Basic Science, Daejeon 305-701, South Korea*

*^2^Graduate School of EEWS and NanoCentury KI, KAIST, Daejeon 305-701, South Korea*

Keywords: metal-semiconductor Schottky diode, internal photoemission, double Schottky barriers, hot electron, solar cell, surface plasmon

^*^To whom correspondence should be addressed. E-mail: jeongypark@kaist.ac.kr

**Fitting of the thermionic emission equation for the Schottky barrier height, ideality factor, and series resistance.**

Figure S1. Fitting I–V curves of (a) the Au/Si and (b) the tandem-structured TiO_2_/Au/Si to the thermionic emission equation. After modification from Au/Si to the tandem-structured TiO_2_/Au/Si, slight changes in the Schottky barrier height (SBH), ideality factor (*n*), and series resistance (*Rs*) were observed.

Figure S1 shows the electrical characteristics of the Au/Si and the tandem-structured TiO_2_/Au/Si diodes, which were investigated by fitting the I–V curves to the thermionic emission equation. For thermionic emission over the barrier, the current of the Schottky contacts, as a function of applied voltage, is given by


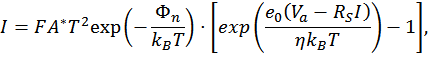


where *F* is the area, *A*^*^ is the effective Richardson constant, Φ_n_ is the Schottky barrier height, η is the ideality factor, and *R_s_*is the series resistance. The effective Richardson constant for silicon is 120 A/cm^2^K.

**Optical properties of the 10 nm thick Au film and TiO_2_ layers**

Figure S2. (a) UV–Vis spectra of the 10 nm thick gold film (black line), the TiO_2_-deposited Au film (red line) and TiO_2_ film on quartz (green line). After deposition of TiO_2_ on the gold film, more incident light penetrates through the TiO_2_/Au film into the silicon. (b) The gap of absorbance spectra obtained by subtracting the absorbance spectrum of the TiO_2_-deposited 10 nm Au film from that of the 10 nm Au film, representing an increased transmitted light toward the silicon.

To confirm the optical properties of the Au film, the TiO_2_-deposited Au film, and the TiO_2_ film on quartz, absorbance spectra were measured using a UV–vis spectrometer. In the case of the TiO_2_-deposited, 10 nm thick Au film on quartz, the absorbance spectrum decreased in visible light compared with the 10 nm thick Au film case, as shown in Figure S2a. However the TiO_2_ film on quartz exhibited a small fraction of the absorption of incident light. Figure S2b shows the gap of absorbance spectra obtained by subtracting the absorbance spectrum of the TiO_2_-deposited, 10 nm thick Au film from that of the 10 nm thick Au film, revealing an increased transmitted light toward the silicon.

**Characteristics of solar cell performance under AM 1.5G**

Figure S3. Characteristics of solar cell performance under AM 1.5G: (a) Fill factor, (b) open-circuit voltage, (c) short-circuit current, and (d) solar cell efficiency.

**Current-Voltage characteristics of Au/TiO_2_ and TiO_2_/Au/TiO_2_ diodes**

Figure S4. I-V curves measured on Au/TiO_2_ diodes before and after annealing and deposition of TiO_2_, indicating that the rectifying behaviors of the diodes were well maintained.
